# Sustainability decision-making in poultry slaughterhouses: A comparative analysis of AHP and fuzzy AHP

**DOI:** 10.1016/j.mex.2025.103193

**Published:** 2025-01-29

**Authors:** Hayati Mukti Asih, Agung Sutrisno, Cynthia E.A. Wuisang, Muhammad Faishal

**Affiliations:** a Department of Industrial Engineering, Faculty of Industrial Technology, Ahmad Dahlan University, Indonesia; b Department of Mechanical Engineering, Faculty of Engineering, Sam Ratulangi University, Indonesia; c Department of Architectural Engineering, Faculty of Engineering, Sam Ratulangi University, Indonesia

**Keywords:** Sustainability, Chicken slaughterhouse, Analytical hierarchy process, Fuzzy AHP, Poultry, Analytic Hierarchy Process (AHP) and Fuzzy Analytic Hierarchy Process (Fuzzy AHP) using different fuzzy numbers for prioritizing sustainable aspects.

## Abstract

The chicken meat industry is vital for global food security and economic growth but faces significant sustainability challenges, especially in balancing economic, environmental, and social aspects. Addressing these challenges in chicken slaughterhouses (CSH) in the Special Region of Yogyakarta, Indonesia, is crucial. This study aims to prioritize criteria for developing strategies to enhance CSH sustainability by comparing the Analytic Hierarchy Process (AHP) and Fuzzy Analytic Hierarchy Process (Fuzzy AHP) using different fuzzy numbers. The findings emphasize the need for a strategy that merges stakeholder engagement, technological innovation, and circular economy principles to advance sustainability. This study fills a research gap by applying multi-criteria decision-making in the poultry industry, which provides a deeper understanding of the robustness and sensitivity of sustainability assessments.

• Employing AHP and Fuzzy AHP with different fuzzy numbers enriches sustainability evaluations by balancing precise judgments and expert uncertainties, which enhancing assessment robustness in the poultry industry.
• Hygiene and sanitation, market competitiveness, and waste minimization are the three highest priorities for sustainable CSH operations across scenarios.
• These findings highlight the need for strategies that integrate stakeholder engagement, innovation, and circular economy principles, addressing a gap in decision-making research for the poultry industry in developing regions.

## Graphical abstract

**Figure:**
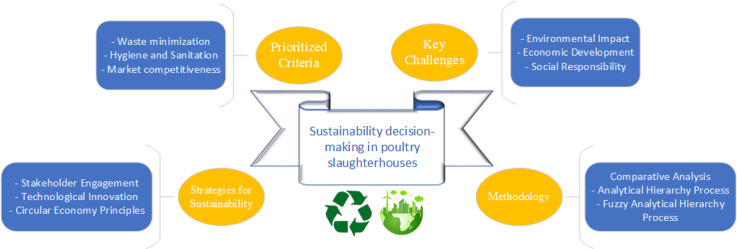

Specifications table

**Table:** 

Subject area:	Engineering
More specific subject area:	Sustainable Poultry Industry
Name of your method:	Analytic Hierarchy Process (AHP) and Fuzzy Analytic Hierarchy Process (Fuzzy AHP) using different fuzzy numbers for prioritizing sustainable aspects.
Name and reference of original method:	Saaty, Thomas L. 1990. ‘How to Make a Decision: The Analytic Hierarchy Process’. *European Journal of Operational Research* 48 (1): 9–26. https://doi.org/10.1016/0377–2217(90)90057-I. Chang, Da Yong. 1996. ‘Applications of the Extent Analysis Method on Fuzzy AHP’. *European Journal of Operational Research* 95 (3): 649–55. https://doi.org/10.1016/0377–2217(95)00300–2.
	Ekmekcioğlu, Ömer, Kerim Koc, and Mehmet Özger. 2021. ‘District Based Flood Risk Assessment in Istanbul Using Fuzzy Analytical Hierarchy Process’. *Stochastic Environmental Research and Risk Assessment* 35 (3): 617–37. https://doi.org/10.1007/s00477–020–01924–8. Coffey, Laura, and David Claudio. 2021. ‘In Defense of Group Fuzzy AHP: A Comparison of Group Fuzzy AHP and Group AHP with Confidence Intervals’. *Expert Systems with Applications* 178 (March): 0–1. https://doi.org/10.1016/j.eswa.2021.114970.
Resource availability:	The data are available in this article.

## Background

The growing emphasis on sustainability across industries underscores the need for innovative approaches to address complex challenges, particularly in the food production sector [1]. Within this realm, the chicken meat industry emerges as a critical player, contributing significantly to global food security and economic growth [2,3]. However, this industry, especially its chicken slaughterhouse (CSH) operations, faces significant challenges in balancing economic profitability with environmental stewardship and social equity [4]. These challenges have driven researchers and practitioners to seek robust methodologies for sustainability assessment and prioritization.

In the context of chicken slaughterhouses, sustainability is a multifaceted issue encompassing economic, social, and environmental dimensions. Economically, factors such as manufacturing costs, productivity, investment cost, and market competitiveness are essential for the industry's viability [[5], [6], [7]]. Socially, workplace safety, customer complaints, employee well-being, training for workers, working conditions; and customer satisfaction are vital for maintaining a balanced relationship between internal stakeholders and the broader community [[8], [9], [10], [11], [12]]. Environmentally, the industry grapples with issues such as waste management, resource optimization, and pollution control [13,14]. Addressing these interconnected dimensions requires comprehensive strategies that harmonize competing priorities.

Motivated by the necessity to prioritize these diverse criteria effectively, this study employs and compares the Analytical Hierarchy Process (AHP) and its extension, Fuzzy Analytical Hierarchy Process (Fuzzy AHP), to effectively prioritize diverse criteria in sustainability assessments. AHP provides a systematic framework for decision-making through pairwise comparisons, offering valuable insights into ranking priorities [15]. However, its reliance on precise numerical judgments limits its ability to account for human cognitive nuances and the uncertainty inherent in subjective evaluations [16,17]. To address these limitations, Fuzzy AHP integrates fuzzy set theory, allowing decision-makers to express preferences using ranges or linguistic terms, thus better capturing ambiguity and variability in complex scenarios [18,19]. The methodologies differ notably in their treatment of uncertainty: AHP assumes certainty in inputs, while Fuzzy AHP uses fuzzy logic with judgments expressed as ranges, such as ``between 3 and 4″ [20,21]. AHP employs a linear additive model with crisp comparisons, whereas Fuzzy AHP uses fuzzy sets and triangular fuzzy numbers, offering greater flexibility [22,23]. Furthermore, Fuzzy AHP is more tolerant of inconsistencies and excels in qualitative assessments, though its computational complexity is higher compared to AHP [24,25].

The motivation behind adopting these methodologies lies in their ability to structure and simplify the decision-making process for sustainability assessment in CSH operations. By comparing AHP and Fuzzy AHP, this research aims to empathize the strengths and limitations of each approach, offering a dual perspective on sustainability prioritization. This methodological comparison is particularly relevant in Special Region of Yogyakarta, where CSH operators face unique regional challenges, including resource constraints, regulatory demands, and cultural considerations. Therefore, this study aims to develop a sustainability framework tailored to the unique challenges of the Yogyakarta region, bridging theoretical concepts and practical applications. By utilizing AHP and Fuzzy AHP, it provides actionable insights for policymakers and practitioners, enabling informed and resilient decision-making in the CSH sector.

## Method details

The poultry industry faces increasingly complex sustainability challenges, which require advanced decision-making tools to address effectively. It balances conflicting sustainability goals, such as economic. environment, and social, as demonstrated in Table 1. Some previous researches have shown a rising interest in sustainability across many sectors, with a focus on using these sophisticated methods to assess and adopt more sustainable practices.

**Table 1:** Related studies focused on sustainability goals.

Author	Sustainability dimensions			Proposed method	Case study
	Economy	Social	Environment		
[25]			√	Fuzzy-AHP, F-TOPSIS, F-GRA	Green new product development
[26]	√	√	√	Hybrid MCDM/MCDA methods	Transportation networks
[27]	√	√	√	Fuzzy-AHP, Shannon entropy	Sustainable development of energy sector in India
[35]	√	√	√	Fuzzy PROMETHEE, Fuzzy MAIRCA, Fuzzy FUCOM	Logistics platform location in Sfax, Tunisia
[28]	√			AHP-TOPSIS	Pharmaceutical supply chain
[29]	√	√	√	Fuzzy Additive Preference Programming	Sustainable management system in rubber factory
[36]	√	√	√	Spherical Fuzzy AHP, CoCoSo	Sustainable chemical supplier selection in Vietnam
[31]	√	√		Fuzzy AHP, COPRAS	Circular supplier selection
[32]			√	Fuzzy-AHP, Fuzzy-TOPSIS, Fuzzy-ELECTRE	Sustainable campus design
[33]	√	√	√	Fuzzy AHP, QFD	Sustainability evaluation of port expanding locations
[34]	√	√	√	Fuzzy DEMATEL, Fuzzy DANP	Sustainable social rental housing
Proposed research	√	√	√	AHP and Fuzzy AHP	Sustainable CSH

In 2021, Ayağ [25] focused on the environmental dimension of green new product development by applying Fuzzy-AHP, F-TOPSIS, and F-GRA to enhance eco-friendly designs. Broniewicz and Ogrodnik [26] took a holistic approach to transportation networks by integrating economic, social, and environmental factors using hybrid MCDM/MCDA methods, ensuring a balanced and sustainable development. Similarly, Saraswat and Digalwar [27] applied Fuzzy-AHP and Shannon entropy to explore sustainable energy sector development in India, tackling the challenges of renewable energy adoption. Ayadi et al. addressed logistics platform location in Tunisia, employing Fuzzy PROMETHEE, Fuzzy MAIRCA, and Fuzzy FUCOM to optimize location decisions based on sustainability criteria.

In 2022, Haji et al. [28] focused on the pharmaceutical supply chain with an emphasis on economic sustainability, applying AHP-TOPSIS to streamline supply chain processes. In contrast, Panjavongroj and Phruksaphanrat [29] took a broader approach to sustainable management in the rubber industry, using Fuzzy Additive Preference Programming to consider economic, social, and environmental dimensions in factory operations. Wang et al. [30] used Spherical Fuzzy AHP and CoCoSo to evaluate chemical supplier selection in Vietnam, ensuring sustainable sourcing by balancing cost, social impact, and environmental concerns. Perçin [31] examined circular supplier selection by applying Fuzzy AHP and COPRAS, emphasizing economic efficiency and social responsibility in the context of circular economy practices.

By 2023, the emphasis on sustainability had broadened significantly. Kalawi et al. [32] focused on sustainable campus design, primarily addressing environmental sustainability through the use of Fuzzy-AHP, Fuzzy-TOPSIS, and Fuzzy-ELECTRE to develop eco-friendly campus models. Wang [33] investigated suitable locations for port expansions, incorporating economic, social, and environmental sustainability dimensions by utilizing Fuzzy AHP and QFD to support sustainable growth in port facilities. Similarly, Jiang et al. [34] addressed sustainable social rental housing, applying Fuzzy DEMATEL and Fuzzy DANP to create affordable, socially inclusive, and environmentally conscious housing solutions. these studies highlight the growing significance of embedding sustainability into decision-making processes across diverse industries, ranging from logistics and supply chains to campus design and housing. Fuzzy-based methods enable researchers to manage the inherent uncertainties in sustainability assessments, which providing a well-rounded approach to addressing economic, social, and environmental challenges. These efforts emphasize the necessity for multi-criteria decision-making tools to handle the complexities of sustainability, which promoting long-term viability across various sectors.

Although sustainability in the poultry industry has been widely researched and MCDM methods applied across various sustainability contexts, a significant gap exists in the literature concerning the application and comparison of AHP and Fuzzy AHP specifically for prioritizing sustainability criteria within CSH. Moreover, studies focusing on the Indonesian context, particularly the Special Region of Yogyakarta Province, remains limited.

Our study aims to address this gap by:

1. Applying a comparative analysis of AHP and Fuzzy AHP in evaluating sustainability priorities in CSH operations, providing a comprehensive comparison of these methods in this specific context.
2. Focusing on the unique sustainability challenges and priorities in the Special Region of Yogyakarta Province, Indonesia, contributing to the geographical diversity of sustainability research in the poultry industry.
3. Developing a framework for sustainability assessment in CSH that integrates economic, environmental, and social dimensions, tailored to the local context but with potential broader applicability.

This study employed a multi-stage methodology to prioritize criteria for developing strategies to enhance the sustainability of CSHs in the Special Region of Yogyakarta Province, Indonesia. Our approach focused on economic, social, and environmental principles, utilizing and comparing both the AHP and Fuzzy AHP methods.

**Fig. 1:**
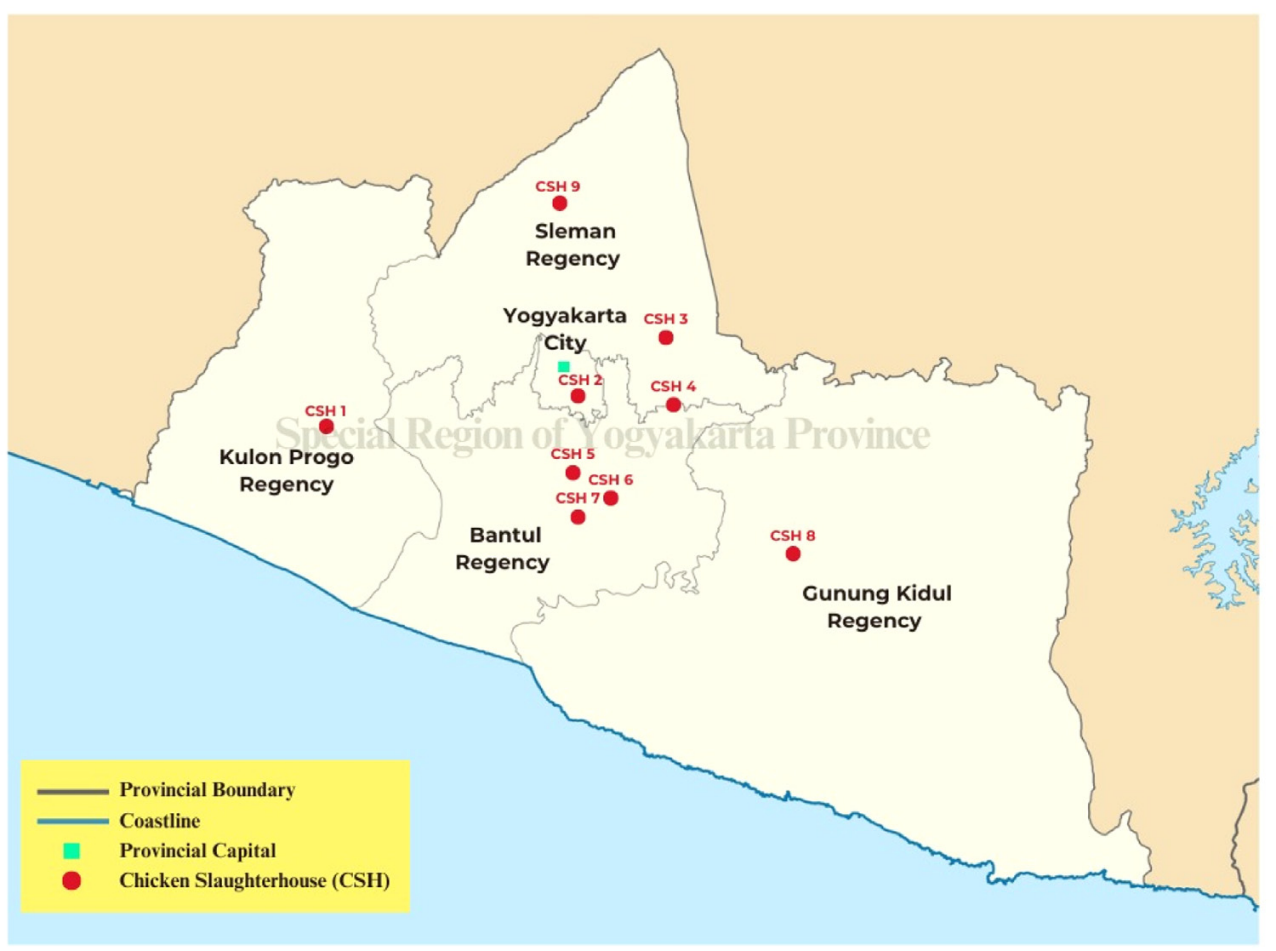
CSH distribution in the special region of Yogyakarta Province, Indonesia.

Data collection was carried out through a combination of questionnaires and semi-structured interviews with key stakeholders in the CSH industries of Special Region of Yogyakarta Province, which include five regencies/cities such as consisting of the Regencies of Gunungkidul, Bantul, Kulon Progo, Sleman, and the City of Yogyakarta (as shown in Fig. 1). Data were collected from nine experts through purposive sampling, ensuring diversity in the perspectives of CSH managers and owners. Experts provided pairwise comparisons for criteria within economic, social, and environmental dimensions. The Fuzzy AHP method used triangular fuzzy numbers to account for uncertainties in expert judgments, following the methodology of [[37], [38], [39]]. To ensure validity, a consistency ratio was calculated for the AHP pairwise comparisons.

To tackle the complex challenge of advancing sustainability in CSH, this study introduces an integrated approach combining AHP with fuzzy set theory, forming a robust Fuzzy AHP methodology. This section outlines the theoretical underpinnings and practical application of the proposed solution.

While AHP offers a robust foundation for decision-making, it may be limited when addressing the uncertainty and vagueness commonly found in real-world situations. To overcome this, the research incorporates fuzzy set theory, introduced by Lotfi A. Zadeh in 1965. Fuzzy set theory expands on classical set theory by permitting partial membership in a set, creating a mathematical structure to represent and handle imprecise or ambiguous information.

In sustainability assessments, where numerous criteria are qualitative and open to interpretation, fuzzy set theory provides a refined approach to capturing expert insights and stakeholder preferences. It enables the use of linguistic terms to express judgments, which are subsequently translated into fuzzy numbers for computational analysis. Combining AHP with fuzzy set theory results in Fuzzy AHP, a robust method that merges the structured framework of AHP with fuzzy logic's capacity to manage uncertainty. This hybrid approach retains the intuitive and straightforward nature of AHP while enhancing its ability to address the imprecision and ambiguity characteristic of human judgment.

The proposed solution employs Fuzzy AHP to evaluate and prioritize sustainability initiatives in CSH. This approach enables decision-makers to articulate comparative judgments using linguistic terms, which are subsequently converted into triangular fuzzy numbers for analysis. This approach not only accommodates the uncertainty in the decision-making process but also provides a more realistic representation of the problem at hand.

**Fig. 2:**
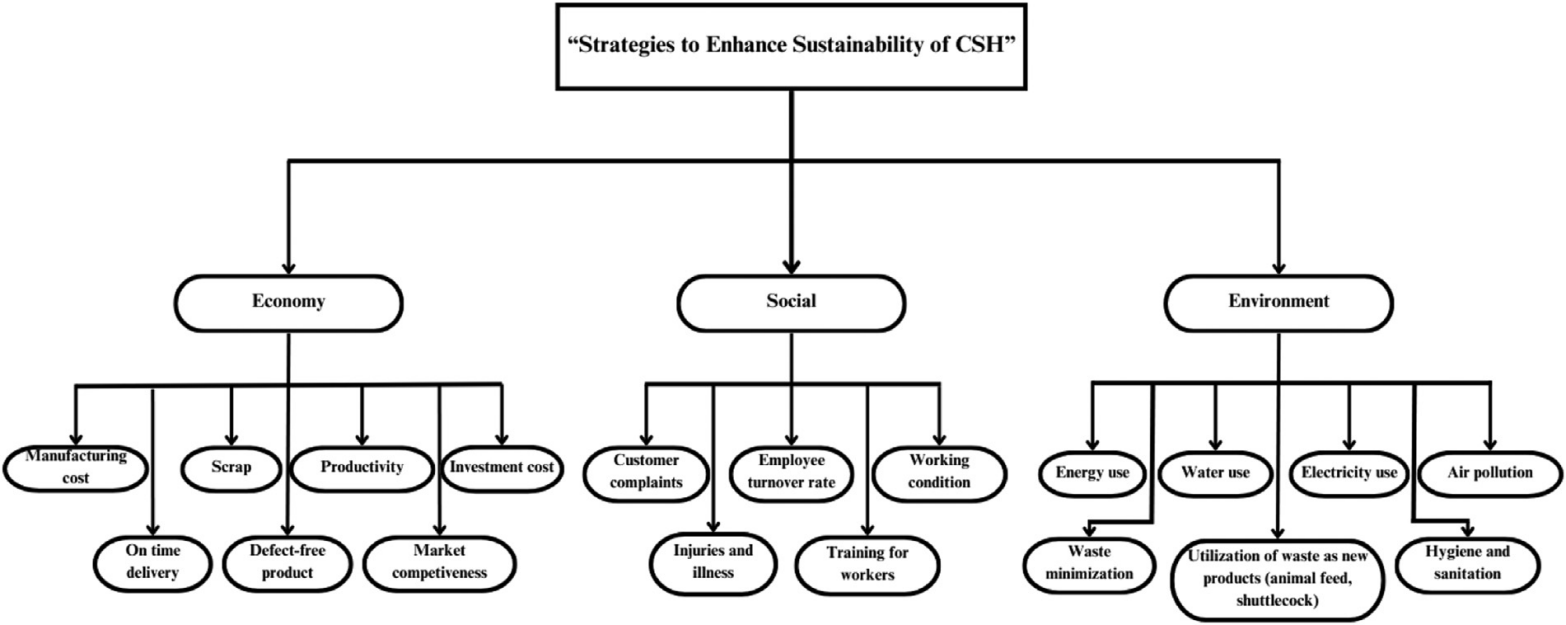
Proposed hierarchical framework.

The application of Fuzzy AHP to enhance sustainability in chicken slaughterhouses follows a structured process:

1. Hierarchy Construction: We begin by constructing a decision hierarchy that decomposes the sustainability goal into three main criteria: economic, environmental, and social aspects. These criteria were structured into a hierarchical framework (refer to Fig. 2), with strategies to enhance the sustainability of CSH as the overall goal, the three aspects as the second level, and the sub-criteria as the third level. This hierarchical structure formed the basis for both the AHP and Fuzzy AHP analyses. Each of these is further divided into sub-criteria as presented in Table 2.

**Table 2:** Criteria and Sub criteria of strategies to enhance the sustainability in CSH.

Criteria	Sub-criteria	Refs.
Economy (ECO)	Manufacturing cost (MC)	[40]
	On-time delivery (OTD)	[41]
	Productivity (PRD)	[42]
	Scrap (SCP)	[42]
	Defect-free product (DFP)	[42]
	Market competitiveness (MKT-C)	[43]
	Investment cost (IC)	[44]
Social (SOC)	Customer complaints (CC)	[40]
	Injuries and illness (II)	[40]
	Employee turnover rate (ETR)	[45]
	Training for workers (TFW)	[46]
	Working conditions (WC)	[47,48]
Environment (ENV)	Energy use (EU)	[41]
	Water use (WU)	[41]
	Electricity use (ELU)	[49]
	Waste minimization (WM)	[40]
	Hygiene and sanitation (HS)	[42,50,51]
	Air pollution (AP)	[46]
	Utilization of waste as new products (e.g., animal feed, shuttlecock) (UWP)	[52]
2. Fuzzy Pairwise Comparisons: Experts conduct pairwise comparisons between criteria and sub-criteria using linguistic terms, which are then converted into triangular fuzzy numbers (TFN). TFN is defined by l,m,u which means l is the lower bound (the least possible value), m is the middle value (the most likely value), and u is the upper bound (the largest possible value), as shown in Fig. 3.

**Fig. 3:**
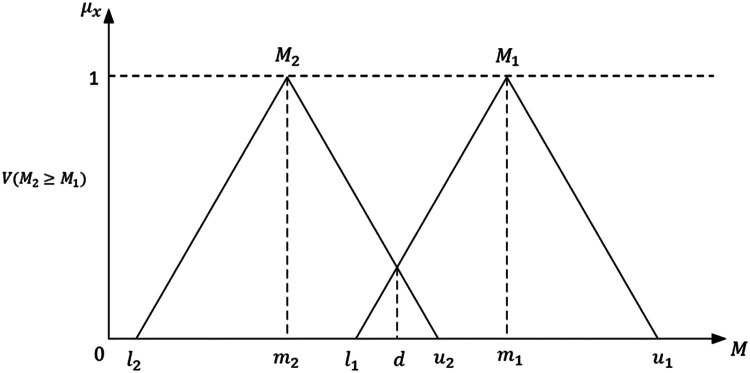
Triangular fuzzy number (TFN).

This research involves three scenarios for comparing different TFNs. In Scenario 1, Saaty (1990)’s AHP scale is employed. Scenario 2 utilizes TFN following the methodology of [37]. Scenario 3 adopts the approach proposed by [38,39]. The results from these scenarios are analyzed to determine the priority of the criteria and sub-criteria in developing strategies to enhance the sustainability of CSH (Table 3).

3. Fuzzy Synthetic Extent Calculation: Using the fuzzy pairwise comparison matrices, we calculate the fuzzy synthetic extent ($S^{*}$) for each criterion and sub-criterion. This step involves fuzzy arithmetic operations to aggregate the fuzzy values. Let $X=\{x_{1},\;x_{2},\ldots ,\;x_{n}\}$ be an object set, and $U=\{u_{1},\;u_{2},\ldots ,\;u_{n}\}$ be a goal set. Based on Chang [53]’s extent analysis method, each object is evaluated individually, and the extent analysis for each goal, $g_{i}$, is conducted accordingly. As a result, m extent analysis values can be derived for each object, represented by the following symbols:

(1) $$M_{g}^{1},\;M_{g}^{2},\ldots ,M_{g}^{m},\;i=1,2,\ldots ,n$$

where all the $M_{g}^{1}\;\left(j=1,2,\ldots ,m\right)$ are TFNs whose parameters are l,m,u. These values represent the minimum possible value, the most possible value, and the maximum possible value, respectively. A TFN is denoted by l,m,u. The steps of Chang's extent analysis can be outlined as follows:

*Step 1*. The fuzzy synthetic extent value for the i-th object is defined as follows:
  (2) $$S_{i}=\sum_{j=1}^{m}M_{gi}^{j}\;⊗{\left[\sum_{i=1}^{n}\sum_{j=1}^{m}M_{gi}^{j}\right]}^{-1}$$

**Table 3:** Linguistic variables corresponding to the Saaty 1–9 scale and fuzzy triangular scale.

Linguistic variables	AHP	Fuzzy AHP	
	Scenario 1 by Saaty [15]	Scenario 2 (Adapted from Chang [37])	Scenario 3 (Adapted from Ekmekcioğlu et al. [39]; Coffey & Claudio [38])
Equally important	1	(1,1,1)	(1,1,1)
Intermediate value	2	(1/2,1,3/2)	(1,2,3)
Moderately important	3	(1,3/2,2)	(2,3,4)
Intermediate value	4	(3/2,2,5/2)	(3,4,5)
Important	5	(2,5/2,3)	(4,5,6)
Intermediate value	6	(5/2,3,7/2)	(5,6,7)
Very important	7	(3,7/2,4)	(6,7,8)
Intermediate value	8	(7/2,4,9/2)	(7,8,9)
Extremely important	9	(4,9/2,9/2)	(9,9,9)

To calculate $\sum_{j=1}^{m}M_{gi}^{j}$ perform the fuzzy addition of the m extent analysis values for a given matrix, as follows:

(3) $$\sum_{j=1}^{m}M_{gi}^{j}=\left(\sum_{j=1}^{m}l_{j},\sum_{j=1}^{m}m_{j},\sum_{j=1}^{m}u_{j}\right),\;i=1,2,\ldots ,n$$

Then, to calculate ${\left[\sum_{i=1}^{n}\sum_{j=1}^{m}M_{gi}^{j}\right]}^{-1}$, perform the fuzzy addition operation for the values of $M_{gi}^{j}\left(j=1,2,\ldots ,m\right)$, as follows:

(4) $$\sum_{i=1}^{n}\sum_{j=1}^{m}M_{gi}^{j}=\left(\sum_{i=1}^{n}\sum_{j=1}^{m}l_{j},\sum_{i=1}^{n}\sum_{j=1}^{m}m_{j},\sum_{i=1}^{n}\sum_{j=1}^{m}u_{j}\right)$$

Next, calculate the inverse of the vector as follows:

(5) $${\left[\sum_{i=1}^{n}\sum_{j=1}^{m}M_{gi}^{j}\right]}^{-1}=\left(\frac{1}{\sum_{i=1}^{n}u_{i}},\;\frac{1}{\sum_{i=1}^{n}m_{i}},\frac{1}{\sum_{i=1}^{n}l_{i}}\right)$$

*Step 2*. The degree of possibility of $M_{2}=\left(l_{2},m_{2},u_{2}\right)$ being greater than or equal to $M_{1}=\left(l_{1},m_{1},u_{1}\right)$ is defined as:
  (6) $$V\left(M_{2}\geq M_{1}\right){=}_{\;\text{yx}}^{sup}\left[\text{min}(\mu_{M_{1}}\left(x\right),\mu_{M_{2}}\left(y\right)\right]$$

This can also be expressed equivalently as follows:

$$V\left(M_{2}\geq M_{1}\right)=hgt\left(M_{1}\cap M_{2}\right)=\mu_{M_{2}}\left(d\right)$$

(7) $$=\left\{ \begin{array}{c} 1,\;\text{if}\;m_{2}\geq m_{1}\\ 0,\;\text{if}\;l_{1}\geq u_{2}\\ \frac{l_{1}-u_{2}}{\left(m_{2}-u_{2}\right)-\left(m_{1}-l_{1}\right)},\;\text{otherwise} \end{array}\right.$$

where d represents the ordinate of the highest intersection point D between $\mu_{M_{1}}$ and $\mu_{M_{2}}$ (refer to Fig. 3). In order to compare $M_{1}$ and $M_{2}$, we require the values of both $V(M_{1}\geq M_{2})$ and $V(M_{2}\geq M_{1})$.

*Step 3*. The degree of possibility that a convex fuzzy number M is greater than k convex fuzzy numbers $M_{i}$ (with i=1,2,…,k) can be described as:
  (8) $$V\left(M\geq M_{1},M_{2},\ldots ,M_{k}\right)=V\left[\left(M\geq M_{1}\right)\;\text{and}\;\left(M\geq M_{2}\right)\;\text{and},\ldots ,\text{and}\;\left(M\geq M_{k}\right)\right]=\begin{array}{c} Min\;V\\ i \end{array}\begin{array}{c} \left(M\geq M_{i}\right) \end{array},\;i=1,2,3,\ldots ,k$$

Assume that

(9) $$d^{\prime }\left(A_{i}\right)=\begin{array}{c} Min\;V\\ i \end{array}\begin{array}{c} \left(S_{i}\geq S_{k}\right) \end{array}$$

For k=1,2,…,n;k≠i. The weight vector is then represented as:

(10) $$W^{\prime }={\left(d^{\prime }\left(A_{1}\right),\;d^{\prime }\left(A_{2}\right),\ldots ,d^{\prime }\left(A_{n}\right)\right)}^{T}$$

where $A_{i}\left(i=1,2,\ldots ,n\right)$ are n elements.

*Step 4*. Through normalization, the normalized weight vectors are:
  (11) $$W={\left(d\left(A_{1}\right),\;d\left(A_{2}\right),\ldots ,d\left(A_{n}\right)\right)}^{T})$$
  Where W represents a non-fuzzy number.
*Step 5.* Priority ranking. Based on the final weights, it ranks the sustainability initiatives and identify the most critical areas for improvement in the CSH.

## Method validation

This section presents the findings from our comparative analysis of AHP and Fuzzy AHP methods in prioritizing sustainability criteria for CSHs in the Special Region of Yogyakarta Province, Indonesia. We discuss the results within the context of economic, social, and environmental sustainability, and explore their implications for developing effective strategies in the CSH industry.

### Criteria prioritization within sustainability aspects

**Fig. 4:**
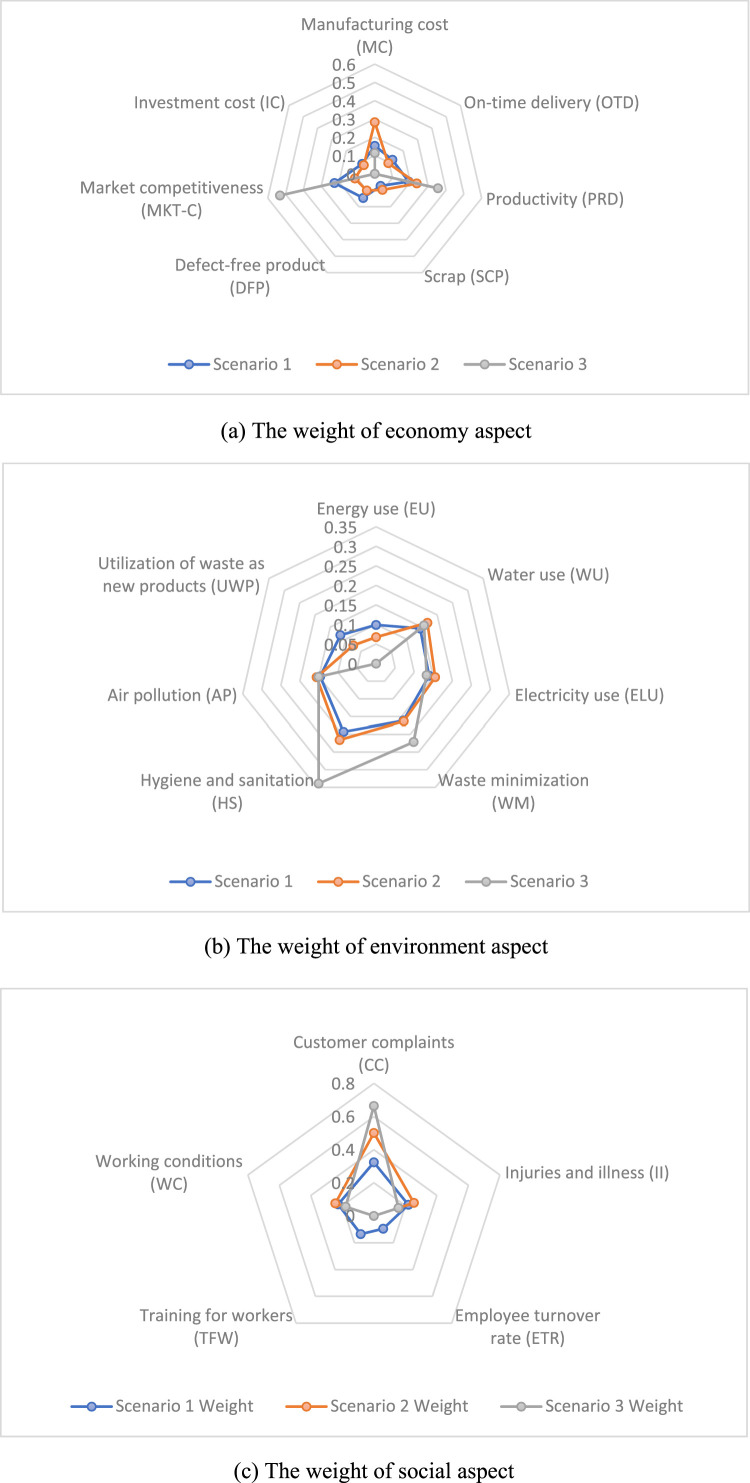
The weight of sustainability aspects.

This subsection examines the prioritization of criteria within the three fundamental aspects of sustainability: economic, environmental, and social. The analysis employs both traditional AHP and Fuzzy AHP methodologies to provide a comprehensive understanding of the prioritization. The results from three distinct scenarios are presented in Fig. 4, each offering unique insights into the relative importance of various criteria within the sustainability framework of CSH in the Special Region of Yogyakarta Province.

For the economic aspect, the data reveals that manufacturing cost, productivity, and market competitiveness are key indicators that vary significantly across scenarios. In Scenario 3, market competitiveness emerges as the most critical factor with a weight of 0.5321, showing its dominant role in ensuring long-term economic viability. Productivity also shows an increasing trend from Scenario 1 to Scenario 3, emphasizing its importance in a sustainable business model. Scenario 1 places higher emphasis on manufacturing costs, which gradually become less significant in later scenarios.

The environmental criteria highlight the importance of energy, water, and waste management. Scenario 3 demonstrates a more focused approach to waste minimization and hygiene, with high values of 0.2211 and 0.3390, respectively. These factors play a critical role in the overall sustainability of the housing model, as minimizing environmental impact is crucial for long-term viability. Scenario 2 shows a balanced approach to water and electricity use, but Scenario 3 appears to prioritize waste management as a strategy to improve environmental performance. The utilization of waste as new products, which shows zero weight in Scenario 3, might indicate a shift towards other waste minimization strategies, such as hygiene and sanitation measures.

From the social perspective, Scenario 3 highlights customer complaints as a significant issue, with a weight of 0.6641, suggesting that addressing customer satisfaction is vital for the success of CSH. While injuries and illness show decreasing significance in Scenario 3, the data suggests that worker training and improving working conditions remain critical for fostering a sustainable and socially responsible housing model. The absence of investment in worker training in Scenario 2 and Scenario 3, however, raises concerns about long-term social sustainability, as continuous education and training are key to improving the overall quality of life for workers and ensuring long-term social cohesion within the slaughterhouse industry.

A comparative analysis of the global ranks across all scenarios is presented in Table 4. It highlights the consistencies and discrepancies in the prioritization of sustainability aspects. This comparison serves to validate the robustness of the findings while also illuminating the potential impact of methodological choices on the final rankings.

**Table 4:** Global ranks of sustainability aspects across all scenarios.

Criteria	Sub criteria	Rank		
		Scenario 1	Scenario 2	Scenario 3
Economy	Manufacturing cost (MC)	9	7	8
	On-time delivery (OTD)	13	12	9
	Productivity (PRD)	4	8	6
	Scrap (SCP)	18	13	10
	Defect-free product (DFP)	10	11	11
	Market competitiveness (MKT-C)	2	2	3
	Investment cost (IC)	16	14	12
Environment	Energy use (EU)	15	10	13
	Water use (WU)	7	3	4
	Electricity use (ELU)	8	6	7
	Waste minimization (WM)	3	4	2
	Hygiene and sanitation (HS)	1	1	1
	Air pollution (AP)	6	5	5
	Utilization of waste as new products (UWP)	11	9	14
Social	Customer complaints (CC)	5	15	15
	Injuries and illness (II)	14	16	16
	Employee turnover rate (ETR)	19	17	17
	Training for workers (TFW)	17	18	18
	Working conditions (WC)	12	19	19

## Discussion

The analysis includes three different scenarios: Scenario 1 applies traditional AHP, while Scenarios 2 and 3 utilize Fuzzy AHP with varying fuzzy number representations. This multi-scenario approach provides valuable insights into how different methodological choices can impact the prioritization of sustainability aspects within chicken slaughterhouses.

Hygiene and Sanitation (HS) consistently emerge as the highest priority for sustainable CSH operations across all scenarios, emphasizing their vital role in ensuring food safety and regulatory adherence. Numerous studies have shown the substantial impact of proper sanitation practices in reducing contamination risks. For example, [54] found that inadequate hygiene and sanitation in traditional chicken slaughterhouses were significant contributors to microbial contamination, particularly in raw material handling and personal hygiene practices. Similarly, [55] demonstrated that disinfectants like peracetic acid and quaternary ammonia, used on cutting room surfaces in poultry slaughterhouses, effectively reduced bacterial contamination, including Escherichia coli and Staphylococcus aureus. Studies in Algeria [56] and Burkina Faso [9] further revealed the ongoing challenges of poor hygiene, leading to bacterial contamination in slaughterhouses [57]. Highlighted that inadequate cleaning practices in Sweden resulted in the persistence of pathogens such as Campylobacter jejuni and Listeria monocytogenes. These findings collectively underscore the essential need for stringent hygiene protocols, infrastructure enhancements, and comprehensive employee training to secure the long-term safety and sustainability of CSH operations.

Market Competitiveness (MKT-C) emerges as another high-priority sub criteria, ranking second in Scenarios 1 and 2, and third in Scenario 3. This consistency highlights the significant role of economic viability in sustainable CSH operations. This aligns with broader studies that emphasize the importance of balancing sustainability and competitiveness in the poultry industry [58]. Highlight how incorporating Industry 4.0 technologies can enhance competitiveness while maintaining sustainability, particularly in Brazilian poultry operations. Similarly, [59] explore how strategic pricing and feed cost improvements are key to competitiveness in the South African broiler industry, despite market volatility. The balance between sustainability, such as animal welfare and environmental impact, and competitiveness is also discussed by [60] in the context of Dutch poultry operations. Furthermore, [6] highlight the significant impact of global crises like the COVID-19 pandemic on production costs, emphasizing the ongoing challenge of maintaining competitiveness in fluctuating global markets. Together, these insights underscore the importance of integrating competitiveness with sustainability principles to ensure long-term success in CSH operations.

Interestingly, the rankings of environmental sub criteria exhibit some variations across scenarios. Waste Minimization (WM), for instance, ranks third, fourth, and second in Scenarios 1, 2, and 3, respectively. This fluctuation suggests that while waste management is consistently recognized as important, its relative priority may be influenced by the specific evaluation methodology used. Studies highlight the critical environmental risks posed by improper waste management, such as wastewater discharge without treatment, which contains harmful pollutants [61]. Various solutions have been explored to mitigate these risks, including natural additives like moringa seed powder to improve wastewater quality [62] and advanced treatment technologies like GAS-SBR for reducing organic pollutants [63]. Additionally, [64] emphasize the sustainable reuse of poultry waste as biofertilizers, enhancing crop energy efficiency while minimizing pollution. A broader review by [65] also outlines sustainable waste management techniques to mitigate the environmental footprint of poultry abattoirs. These findings highlight the critical role of effective waste management in achieving sustainability in CSH operations, even as its relative priority may vary depending on context.

This comprehensive analysis of global ranks provides valuable insights for stakeholders in the CSH industry. It not only identifies key priority areas for sustainable operations but also illustrates how different evaluation methodologies can influence sustainability assessments. The findings can guide strategic decision-making, resource allocation, and policy formulation to enhance the overall sustainability of CSH operations.

By presenting these results, this subsection aims to contribute to a more nuanced understanding of sustainability priorities in the CSH sector, encouraging a balanced approach that considers economic viability, environmental responsibility, and social welfare in the pursuit of sustainable CSH operations.

### Implications for sustainability strategies in CSH

The findings suggest several key strategic focus areas that can be prioritized to enhance sustainability in the CSH industry. These areas aim to address critical challenges and promote long-term environmental, economic, and social sustainability within the industry.

1. Integrated Sustainability Management: Develop a holistic sustainability strategy that balances top-ranking priorities (hygiene, market competitiveness, waste management) with lower-ranked but crucial social aspects.
2. Technology Investment: Focus on technologies that address multiple sustainability aspects simultaneously, such as smart systems that improve hygiene, reduce waste, and enhance productivity.
3. Stakeholder Engagement: Collaborate with local government, environmental agencies, and community organizations to address broader sustainability challenges, particularly in waste management and pollution control.
4. Sustainability Certification: Pursue internationally recognized sustainability certifications to enhance market competitiveness and demonstrate commitment to best practices.
5. Circular Economy Initiatives: Develop partnerships within the agricultural sector to create closed-loop systems, particularly in waste management and resource utilization.
6. Workforce Development: Despite lower rankings, prioritize investments in employee training, working conditions, and retention strategies to ensure long-term operational sustainability.
7. Innovation Hub: Position Yogyakarta as an innovation hub for sustainable CSH practices, encouraging research partnerships with local universities and technology providers.

By focusing on these strategic areas, CSH industry can enhance their sustainability performance, improve market competitiveness, and contribute to the broader sustainable development goals of the region. This approach not only addresses immediate priorities but also lays the groundwork for long-term sustainability in the poultry processing industry.

## Limitations

Not applicable.

## Ethics statements

The work doesn't involve animal or human subject. Additionally, there is no data from social media platforms involved.

## CRediT authorship contribution statement

**Hayati Mukti Asih:** Conceptualization, Methodology, Writing – original draft. **Agung Sutrisno:** Conceptualization, Writing – original draft. **Cynthia E.A. Wuisang:** Conceptualization. **Muhammad Faishal:** Validation.

## Declaration of competing interest

The authors declare that they have no known competing financial interests or personal relationships that could have appeared to influence the work reported in this paper.

## Data Availability

The data that has been used is confidential.
